# Unconventional Maturation of Dendritic Cells Induced by Particles from the Laminated Layer of Larval Echinococcus granulosus

**DOI:** 10.1128/IAI.01959-14

**Published:** 2014-08

**Authors:** Cecilia Casaravilla, Álvaro Pittini, Dominik Rückerl, Paula I. Seoane, Stephen J. Jenkins, Andrew S. MacDonald, Ana M. Ferreira, Judith E. Allen, Álvaro Díaz

**Affiliations:** a Cátedra de Inmunología, Departamento de Biociencias (Facultad de Química) e Instituto de Química Biológica (Facultad de Ciencias), Universidad de la República, Montevideo, Uruguay; b Institute of Immunology and Infection Research, Centre for Immunity, Infection and Evolution, School of Biological Sciences, University of Edinburgh, Edinburgh, United Kingdom

## Abstract

The larval stage of the cestode parasite Echinococcus granulosus causes hydatid disease in humans and livestock. This infection is characterized by the growth in internal organ parenchymae of fluid-filled structures (hydatids) that elicit surprisingly little inflammation in spite of their massive size and persistence. Hydatids are protected by a millimeter-thick layer of mucin-based extracellular matrix, termed the laminated layer (LL), which is thought to be a major factor determining the host response to the infection. Host cells can interact both with the LL surface and with materials that are shed from it to allow parasite growth. In this work, we analyzed the response of dendritic cells (DCs) to microscopic pieces of the native mucin-based gel of the LL (pLL). *In vitro*, this material induced an unusual activation state characterized by upregulation of CD86 without concomitant upregulation of CD40 or secretion of cytokines (interleukin 12 [IL-12], IL-10, tumor necrosis factor alpha [TNF-α], and IL-6). When added to Toll-like receptor (TLR) agonists, pLL-potentiated CD86 upregulation and IL-10 secretion while inhibiting CD40 upregulation and IL-12 secretion. *In vivo*, pLL also caused upregulation of CD86 and inhibited CD40 upregulation in DCs. Contrary to expectations, oxidation of the mucin glycans in pLL with periodate did not abrogate the effects on cells. Reduction of disulfide bonds, which are known to be important for LL structure, strongly diminished the impact of pLL on DCs without altering the particulate nature of the material. In summary, DCs respond to the LL mucin meshwork with a “semimature” activation phenotype, both *in vitro* and *in vivo*.

## INTRODUCTION

The larval stage (hydatid) of the cestode platyhelminth parasite Echinococcus granulosus causes cystic echinococcosis (hydatid disease) in a range of livestock species and also in humans (1). Hydatids are fluid-filled, bladder-like structures that dwell in the parenchyma of internal organs, most commonly the liver and lungs, and can, over years or decades, reach tens of centimeters in diameter (2). Their defining feature is the hydatid wall that surrounds the fluid-filled cavity, composed of a thin inner layer of cells (the germinal layer) and an outer, thick, protective acellular layer (the laminated layer [LL]).

Immunologically, hydatid disease constitutes a striking puzzle, since in spite of the parasite's size, systemic anatomical location, and persistence and the presence of detectable parasite-specific (antibody and proliferative) responses, the parasite normally thrives surrounded by a noninfiltrated host-derived collagen capsule (3). The strong suppressive mechanisms at play in this infection are still undefined, but clues include the high levels of expression of the anti-inflammatory cytokine interleukin 10 (IL-10) by leukocytes near the parasite (4) and the capacity of lymph node cells from infected mice to transfer the suppression of antibody responses to unrelated antigens (5). Importantly, both of these regulatory activities are present in animals in which hydatids are surgically implanted, indicating that exposure to the prehydatid parasite stages is dispensable for induction of these processes. In addition, a strong relative expansion of FoxP3^+^ regulatory T cells in the spleen and blood of infected mice has been recently reported (6).

Healthy hydatids expose the host immune system only to soluble excretory/secretory products (as yet largely uncharacterized) and the massive, carbohydrate-rich LL, which can attain over 2 mm in thickness. With variations, the LL is present in all species of the genus Echinococcus while it is absent from other larval cestodes (2, 3). It is essentially a meshwork of mucins giving rise to an aqueous gel (2, 7). In E. granulosus, but probably not in the other species, the LL additionally contains dispersed nanodeposits of the calcium salt of inositol hexakisphosphate (8–10). The mucin backbones, deduced from the germinal layer transcriptome (2, 11, 12), comprise highly glycosylated domains and short nonglycosylated N-terminal extensions. Certain of these N-terminal extensions bear unpaired cysteine residues, coincident with evidence that disulfide bonds contribute to the LL structure (7). The LL mucin glycans are now largely defined (13, 14).

The LL is widely considered to be the crucial element of larval echinoccocal infections (3, 15, 16), including infection by Echinococcus multilocularis, the causative agent of life-threatening alveolar echinococcosis (1). In addition to protecting the parasite from host immune cells (17), the LL likely contributes to the downregulation of inflammatory responses mounted earlier in infection against the establishing larvae (3, 18, 19). Given the key role played by the innate immune system in coordinating inflammatory responses, the way in which innate immunity decodes, and responds to, LL components is a central piece of the puzzle of larval Echinococcus immunology. The issue has been addressed for the complement system, where triggering of this cascade by the LL is much less effective than that by other E. granulosus materials (3, 20, 21). For innate immune cells, two studies focusing on nitric oxide production by macrophages showed that LL-derived materials inhibit this response in cells stimulated with lipopolysaccharide (LPS) or gamma interferon (IFN-γ) (22, 23).

Immune cells can interact both with the outer surface of the LL and with shed materials, as shedding of LL components is necessary for the hydatid to grow (2). Given the drastic treatments that need to be applied to the E. granulosus LL to achieve solubilization *in vitro* (7), it is most likely that *in vivo* shedding involves particulate, rather than soluble, LL remnants. In experimental mouse infections, periparasitic host macrophages have been observed to phagocytose LL-derived particles (24, 25). Recently, abundant particulate LL-derived materials have been detected in tissue sections from patients infected by E. multilocularis (26). These particles can be found millimeters away from the LL itself, including in lymph nodes draining the infected organ.

We generated particles from the E. granulosus mucin-based gel and analyzed their impact on the activation phenotype of murine dendritic cells (DCs). Our results show that LL particles induce an unconventional “semimature” phenotype in DCs. Surprisingly, LL carbohydrates appear to be dispensable for these effects, while treatments reducing disulfide bonds in the LL materials impaired their ability to induce even this limited maturation state.

## MATERIALS AND METHODS

### Preparation of the pLL.

The starting material for preparation of the mucin-enriched particulate preparation from the LL (pLL) consisted of hydatid walls from macroscopically healthy, fertile (i.e., protoscolex-producing) hydatids. Hydatid walls are in practice equivalent to LL, as the contribution of the very thin germinal layer is negligible (3). The hydatid material used was of Uruguayan origin and was from natural infections in cattle or, where indicated, from mice infected intraperitoneally with protoscoleces. The hydatid walls were extensively washed with phosphate-buffered saline (PBS) and then 2 M NaCl. The walls were then dehydrated and finely ground using a mortar and pestle, followed by grinding between two polished glass plates. The resulting fine powders were carefully rehydrated in pyrogen-free PBS containing 30 mM EDTA (1 ml for every 2 mg dry mass) to extract the calcium Ins*P*_6_ deposits (10), sequentially filtered through 85- and 23-μm gauze, and then extensively washed in pyrogen-free PBS, with centrifuging at 3,000 × *g* for 5 min each time. The dehydration was carried out either with ethanol and acetone as described previously (9) (EA-pLL) or by freeze-drying (FD-pLL). In addition, we tested pLL prepared by a method that did not involve a dehydration step, namely, shredding with a Tissue-ruptor blender (Qiagen) and then applying mild sonication (SS-pLL). The LL dry mass content of each preparation was calculated by freeze-drying and weighing a portion of suspension previously freed from salts by extensive washes with water. Visualization of the preparations under a light microscope was carried out after staining with the lectin PNA labeled with fluorescein (Vector Laboratories) (7). All pLL preparations were kept at 4°C as suspensions in PBS with penicillin-streptomycin. Two different pLL preparations tested negative for endotoxin by the Limulus amebocyte lysate (LAL) method (<0.03 endotoxin units/ml for a dilution equivalent to the maximum pLL dose used with cells). In agreement, neither pLL preparations nor the supernatants of the last washes with PBS mentioned above elicited IL-12p40 or IL-10 from DCs (in excess of the minimal cytokine levels detected for cells incubated with medium alone).

### Treatments applied to pLL.

FD-pLL (from a bovine or mouse host, as indicated in each case) was subjected to a series of treatments. To remove strongly bound antibodies, pLL was suspended in 200 mM glycine-HCl, pH 2, and incubated for 2 h at 50°C with occasional vortexing. To oxidize carbohydrates, pLL was suspended in 50 mM sodium acetate, pH 4.5, containing 10 mM sodium metaperiodate and incubated for 1 h at room temperature with stirring. Then, to reduce and thus stabilize aldehyde groups formed, an equal volume of 200 mM sodium borohydride in water (pH unadjusted) was added, and the suspension was incubated for 30 min at room temperature with stirring. A mock treatment was also applied, in which metaperiodate was omitted from the acetate buffer step but the borohydride reduction was applied as for the oxidative treatment. Destruction of terminal monosaccharides was verified in terms of abrogation of the binding of the plant lectins PNA and RCA1, known to bind the LL sugars (7). For probing in dot blot format, pLL was previously solubilized as described previously (7). For nonspecific proteolysis, pLL was suspended in 20 mM Tris-HCl, pH 8.0, containing 125 μg/ml proteinase type XIV from Streptomyces griseus (pronase; Sigma) and incubated for 18 h at 37°C. For reduction of disulfides, pLL was suspended in 50 mM Tris-HCl, pH 8.0, buffer containing 50 mM dithiotreitol (DTT) and incubated for 1 h at 37°C with occasional stirring. After a single wash with water, pLL was resuspended in 100 mM iodoacetamide (IAA) in the same buffer (to alkylate free thiols) and incubated for 1 h at room temperature in the dark. The pLL suspensions were transferred to pyrogen-free water before all treatments and transferred back into pyrogen-free PBS by extensive washing after the treatments. In the case of proteolytic treatment, it was verified that no proteolytic activity, detectable using the *N*-suc-Ala-Ala-Phe-7-amido-4-methylcoumarin fluorogenic substrate (Sigma), remained after the fourth posttreatment wash.

### BMDC generation.

Murine bone-marrow-derived dendritic cells (BMDCs) were generated by differentiation of bone marrow precursors from 8- to 10-week-old C57BL/6 mice for 10 days in the presence of 20 ng/ml recombinant mouse GM-CSF (PeproTech) as described in reference 27. The cells obtained were between 85 and 95% CD11c^+^. On day 10, cells were plated at 0.4, 1, or 2 million per well in 96-, 48-, or 24-well plates, respectively, and stimuli were added in medium containing 5 ng/ml granulocyte-macrophage colony-stimulating factor (GM-CSF).

### Exposure of BMDCs to pLL and other stimuli.

Doses of pLL and its variants throughout this work are given in terms of μg total dry mass per million cells. These figures are at least an order of magnitude higher than those we would give if we had chosen to express doses in terms of the protein content, since (i) the LL is mostly carbohydrate and (ii) the freeze-drying used to estimate dry mass does not remove all the water from this highly hydrophilic material. When used together with Toll-like receptor (TLR) agonists (10 ng/ml LPS from Escherichia coli O127:B8 [Sigma]; 100 ng/ml Pam3CSK [Invivogen]) or whole heat-killed Propionibacterium acnes (10 μg/ml total dry mass), pLL was added to the cells 1 h before the inflammatory stimulus unless otherwise specified. Cell responses (cytokines in supernatants or expression of cell surface molecules) were assessed 18 h after the last stimulus was added. The blocking antibody against FcγRII/III (CD16/CD32) was from BD and was used at 5 μg/ml. Cytochalasin D was purchased from Sigma and used at 5 μM. Cell viability was analyzed on the basis of exclusion or the LIVE/DEAD dye (Invitrogen) and measured by flow cytometry. BMDCs exposed overnight to 25 μg of FD-pLL (per million cells), whether in the presence or absence of LPS, suffered a significant but minor (approximately 5%) drop in viability, while lower doses of pLL had no effect (see Fig. S1 in the supplemental material). All experiments were carried out with this or a lower dose of pLL.

### Measurement of cell responses.

IL-10, IL-12p70, IL-12/23p40, tumor necrosis factor alpha (TNF-α), and IL-6 were measured in culture supernatants by capture enzyme-linked immunosorbent assay (ELISA) using paired antibodies from BD or R&D. Surface molecules (CD80, CD86, CD40, major histocompatibility complex class II [MHC-II], and CD11c) were quantified by flow cytometry using antibodies conjugated to fluorescein isothiocyanate (FITC), phycoerythrin (PE), peridinin chlorophyll protein (PerCP), and PE-Cy7 (and appropriate isotype controls) from BD or eBioscience. Data were acquired on FacsCalibur or Facs Canto II cytometers and analyzed using the FlowJo package, gating on CD11c^+^ events. When quantifying cell surface molecule expression, the fluorescence levels of isotype control stains were subtracted.

### *In vivo* administration of pLL.

Female 8-week-old C57BL/6 mice were inoculated intraperitoneally (i.p.) with 150 μg of FD-pLL, 15 μg of LPS from E. coli O127:B8 (Sigma), FD-pLL plus LPS, or vehicle (PBS) in 200-μl volumes in all cases. The procedure was approved by the Universidad de la República's Animal Experimentation Committee (CHEA protocol 050510). Either 3 or 18 h later, as indicated, mice were killed, and peritoneal cells were retrieved and analyzed by flow cytometry. DCs were defined as CD19^−^ F4/80^−^ CD11c^+^ MHC-II^+^ cells or simply as CD11c^+^ MHC-II^+^ cells, as the two definitions were found to be clearly equivalent (as shown in Results). The antibodies used were the same as those mentioned above. In addition, peritoneal contents, retrieved by instillation of 1 ml of 0.2% (vol/vol) fetal bovine serum in RPMI, were analyzed for cytokines by ELISA.

### Statistics.

All results were analyzed by one-way analysis of variance (ANOVA), with a Tukey posttest. Overall *P* values are given in the figure legends, and significant differences between treatments/groups are shown in the figures. In addition, for the *in vivo* experiments only, groups were compared across repeated experiments by the restricted maximum-likelihood (REML) method with a Tukey posttest. The REML method is the preferred method for two-way analysis of data when random variations across experiments are expected (28). Hence, for *in vivo* experiments, in addition to the intraexperiment differences, significant differences across repeat experiments are shown in the figures.

## RESULTS

### The LL breaks either parallel or perpendicular to its natural laminations, depending on the pulverization method.

**FIG 1:**
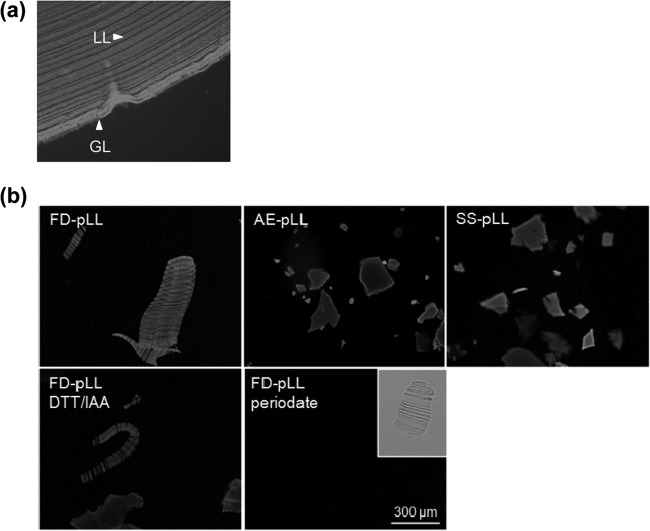
The LL tends to give rise to flat particles upon disruption. Shown are fluorescence microscopy images of the native LL (a paraffin-embedded section from a natural cattle infection) (a) and pLL preparations (b). The images are of pLL prepared by the three methods explained in the text (FD-, EA-, and SS-pLL); note that the laminations present in the native LL are observed only in FD-pLL. Also shown are FD-pLL preparations subjected to reduction/alkylation of disulfides (DTT/IAA) or oxidation of carbohydrate residues with periodate (followed by reduction of aldehydes with borohydride). In both panels, the samples have been labeled with the lectin PNA conjugated to FITC for easier visualization. Periodate oxidation abrogates PNA binding, as expected, but the physical preparation of the material does not change, as can be observed in the clear-field image (inset). The pLL preparations shown are of bovine host origin, except for SS-pLL, which is from a mouse host.

The LL is a physically robust macroscopic structure. Blending the LL in its native (hydrated) form or pulverizing it under liquid nitrogen gave rise mostly to particles far too large for cells to phagocytose. In order to obtain preparations that included particles susceptible to uptake and thereby of similar size to those observed in periparasitic host macrophages *in vivo* (25, 26), we pulverized LL material that had been previously dehydrated. We tried two different dehydration methods, namely, exposing the material to ethanol and then acetone (EA-pLL) and freeze-drying (FD-pLL). The fine powders obtained were rehydrated, yielding aqueous suspensions of particles similar in appearance to the native LL gel. We also generated particles without an intervening dehydration-rehydration step by shredding, followed by sonication (SS-pLL). In spite of the 23-μm-filtering step applied, pLL preparations consistently contained some particles whose size exceeded the cutoff of the filter used (Fig. 1). This was likely due to a tendency of the LL to split into flat and highly flexible particles that could pass through the pores end-on and/or in folded conformations. Whereas particles in EA-pLL or SS-pLL seemed to be generated from individual “laminations,” those in FD-pLL tended to consist of “stacks of laminations.” We hypothesize that the LL has an intrinsic tendency to generate flat particles upon disruption, likely because its strata, or laminations, tend to separate from each other readily. For unknown reasons, this tendency is not apparent when the LL is pulverized after freeze-drying.

### Exposure to pulverized LL alters the cytokine response to TLR agonists of DCs.

**FIG 2:**
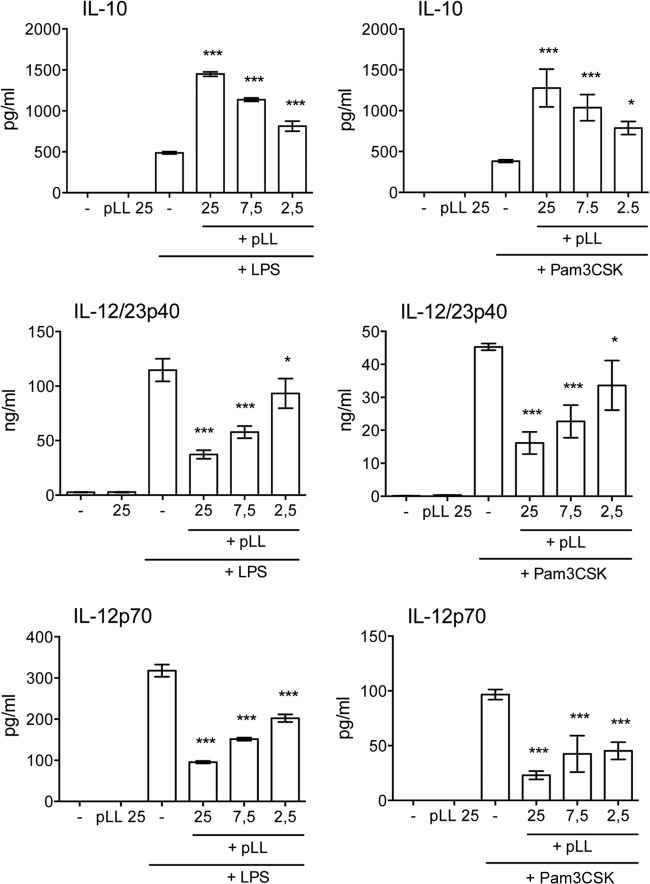
pLL potentiates the production of IL-10 and inhibits that of IL-12/23p40 and IL-12p70 in BMDCs stimulated with TLR agonists. BMDCs were incubated with the doses of FD-pLL indicated (μg) and stimulated with either LPS or Pam3CSK, and cytokine levels in the supernatants were measured. The error bars indicate the standard deviations (SD) of triplicate wells. ANOVA gave overall *P* values of <0.0001 for all the stimulus-response combinations shown. The results shown are representative of at least 6 (LPS) or 3 (Pam3CSK) independent experiments. The asterisks denote differences with respect to stimulation with TLR agonist alone. ***, *P* < 0.001; *, *P* < 0.05.

BMDCs were incubated overnight with different doses of pLL with or without TLR agonists in order to assess whether pLL had the capacity to alter inflammatory processes. Exposure to pLL on its own did not induce secretion of IL-12/23p40, IL-12p70, or IL-10 (Fig. 2), nor did it elicit TNF-α or IL-6 (data not shown). In addition to indicating that LL mucins do not activate DCs in a similar way to TLR agonists, this also shows that the pLL preparation effectively excludes contamination by environmental pyrogens, as independently determined by the LAL method (see Materials and Methods). When the cells were exposed to pLL followed by a TLR4 or a TLR2 agonist (LPS or Pam3CSK, respectively), secretion of IL-12/23p40 and IL-12p70 was strongly inhibited, while that of IL-10 was strikingly elevated in a dose-dependent manner (Fig. 2). The same bias in IL-12 and IL-10 responses was observed when pLL-conditioned DCs were stimulated with a heat-killed preparation of the Gram-positive bacterium P. acnes (data not shown). Levels of TNF-α and IL-6 induced by TLR agonists were not significantly biased by exposure to pLL (data not shown). Similar effects on IL-10 and IL-12/23p40 were observed in bone marrow-derived macrophages (BMDMs) stimulated with LPS or P. acnes (data not shown).

**FIG 3:**
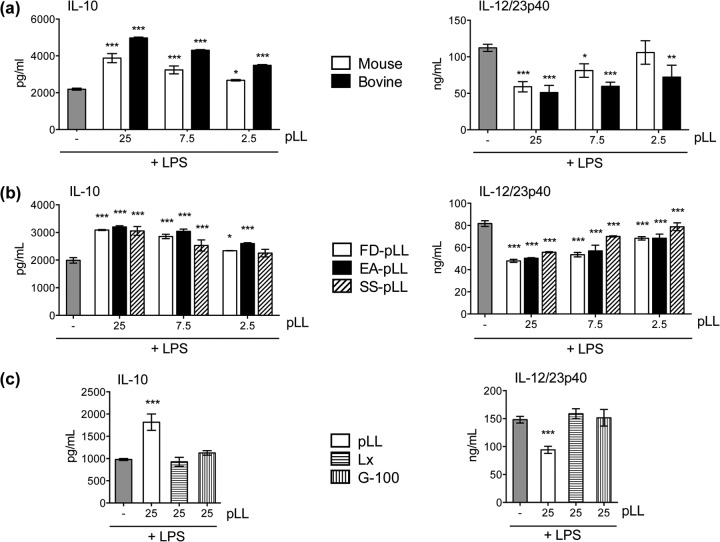
pLL affects BMDC cytokine responses irrespective of the host species origin or the pulverization method used to prepare it; control particles do not show the same activities. BMDCs were incubated with the doses of pLL or control particles indicated (μg) and stimulated with LPS, and levels of IL-10 and IL-12/23p40 in the supernatants were measured. (a) Comparison of FD-pLL prepared from bovine or mouse host materials. (b) Comparison of pLL (from mouse host material) prepared by the three different methods (FD-, EA-, and SS-pLL). (c) Comparison of FD-pLL (bovine host) to (phagocytosable) 0.8-μm polystyrene latex beads coated with bovine serum albumin (BSA) (Lx) or to (nonphagocytosable) Sephadex G-100 beads (G-100). The error bars indicate the SD of triplicate wells. ANOVA gave overall *P* values of <0.0001 (a and b) and <0.001 (c). The results shown are representative of at least 5 (a) or 3 (b and c) individual experiments. The asterisks denote differences with respect to stimulation with LPS alone. ***, *P* < 0.001; **, *P* < 0.01; *, *P* < 0.05.

**FIG 4:**
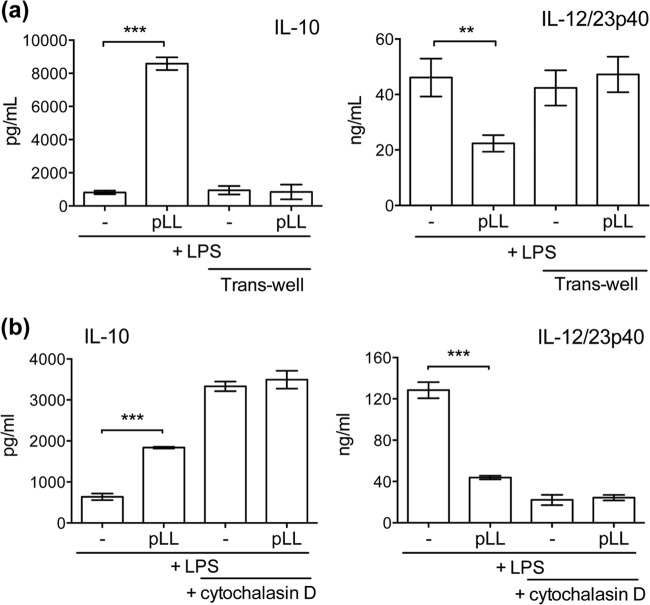
The effects of pLL on BMDC cytokine responses require contact and are not observed in the presence of an actin cytoskeleton inhibitor. BMDCs were incubated with FD-pLL (25 μg) and stimulated with LPS, and cytokine levels in the supernatants were measured. This was carried out comparatively in the absence or presence of transwell inserts (3 μm; pLL was placed below the inserts and the cells above) (a) and in the absence or presence of cytochalasin D (b). The error bars indicate the SD of triplicate wells. ANOVA gave overall *P* values of <0.0001. The results shown are representative of 3 (a) or 5 (b) independent experiments. **, *P* < 0.01; ***, *P* < 0.001.

The effects on BMDC cytokine responses were present in pLL prepared from material derived from two different host species (Fig. 3a), supporting a parasite- rather than host-derived activity. Also, importantly, the effects were present in pLL prepared by the three different methods mentioned above (Fig. 3b). This shows that the activities are present in finely divided LL irrespective of the physical presentation of the particles. It also demonstrates that the activities are not generated as a result of dehydration-rehydration and are not susceptible to denaturation by organic solvents. Because pLL contains both phagocytosable and nonphagocytosable particles, we tested both phagocytosable 0.8-μm polystyrene latex beads and nonphagocytosable Sephadex G-100 (29) to assess if uptake of particles was responsible for the observed results. Neither type of control particle caused biases in IL-10 or IL-12/23p40 responses to LPS (Fig. 3c). The effects of pLL on cytokine responses required contact between the cells and the particles (Fig. 4a). The effects were absent in the presence of the actin polymerization inhibitor cytochalasin D, although interpretation of this result is complicated by the fact that the inhibitor by itself had effects similar to those of pLL (Fig. 4b).

**FIG 5:**
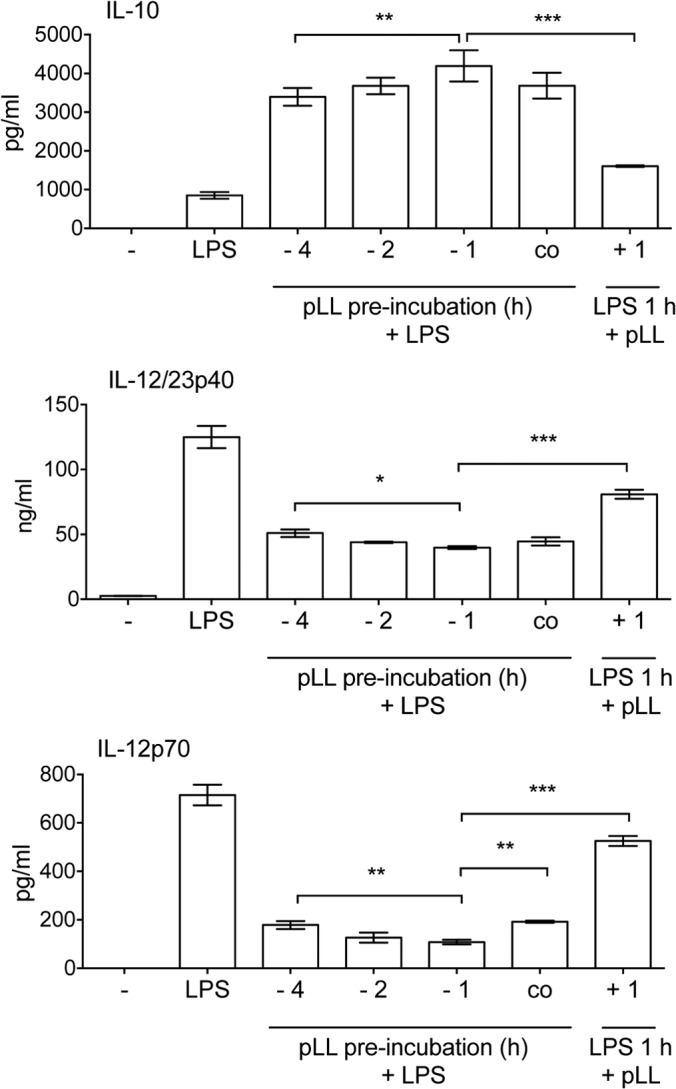
The effects of pLL arise quickly; pLL added after LPS can still shift the response to the TLR agonist. BMDCs were stimulated with LPS alone or with LPS and FD-pLL (25 μg), added at different times with respect to the addition of LPS. IL-10, IL-12/23p40, and IL-12p70 in the supernatants were measured 18 h after LPS addition. The error bars indicate the SD of triplicate wells. ANOVA gave overall *P* values of <0.0001. Only differences in comparison with the conditions used in the previous figures (pLL added 1 h before LPS) are indicated with asterisks. The data are representative of 5 (IL-10), 4 (IL-12/23p40), or 3 (IL-12p70) independent experiments (although in some experiments, the effects of pLL added 4 h before LPS were weaker than those in the experiment shown). ***, *P* < 0.001; **, *P* < 0.01; *, *P* < 0.05.

Our experimental system preincubated BMDCs with pLL, followed by addition of TLR agonists 1 h later. We next analyzed the influence on DC cytokine production of pLL added at different times before or after the addition of LPS. The effects were generally strongest when pLL was added 1 to 2 h before the TLR agonist or at the same time (Fig. 5). Addition of pLL 1 h after the TLR agonist had a weaker, though still discernible, effect. These results indicate that the effects of pLL on cells arise rapidly.

When injected i.p. into mice, pLL by itself did not elicit IL-10 or IL-12/23p40 detectable in peritoneal lavage fluid (see Fig. S2 in the supplemental material), consistent with the *in vitro* results. Also consistent with those results, when coinjected with LPS, pLL caused an important potentiation of the IL-10 response. However, IL-12/23p40 was also potentiated (albeit to a lesser degree than IL-10) rather than inhibited, suggesting that non-DC cell types contribute to the *in vivo* responses to pLL.

In summary, exposure to pLL does not elicit IL-12, IL-10, IL-6, or TNF-α responses from BMDCs but conditions the cells to respond to TLR agonists with an anti-inflammatory cytokine profile.

### Exposure to pLL causes upregulation of CD86 and inhibits upregulation of CD40 in dendritic cells in vitro and in vivo.

**FIG 6:**
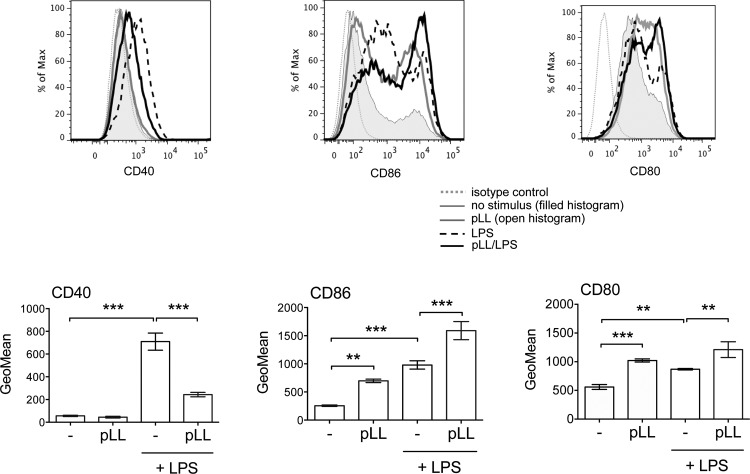
pLL has different effects on CD86 and CD40 expression. BMDCs were incubated with FD-pLL (25 μg) or medium only and then stimulated or not with LPS and analyzed for surface expression of CD86, CD40, and CD80. ANOVA gave overall *P* values of <0.0001 for CD86 and CD40 and <0.001 for CD80. The histograms are representative of results from triplicate wells, and the error bars indicate the SD of these triplicates. The data shown are representative of at least 6 independent experiments, except that effects of pLL on CD80 were not always significant. **, *P* < 0.01; ***, *P* < 0.001.

An assessment of costimulatory molecules showed that incubation of BMDCs with pLL caused a marked increase in the expression of CD86 without an accompanying increase in CD40 (Fig. 6). CD80 was also observed to be upregulated in response to pLL (Fig. 6), but this upregulation was not consistent among different experiments. The levels of MHC-II were unaffected (data not shown). As expected, incubation with LPS caused increased CD86 and CD40 expression. Preincubation with pLL strongly inhibited CD40 upregulation in response to LPS (Fig. 6). Contrasting with this, incubation with pLL before LPS or other TLR agonists caused additive increases in CD86 expression (Fig. 6 and data not shown). Exposure to pLL also tended to potentiate CD80 upregulation (Fig. 6), but again, this was not consistent across experiments. There were no marked effects on MHC-II expression in the presence of LPS (data not shown). The capacity of pLL to upregulate CD86 and inhibit CD40 upregulation was consistently observed with pLL prepared by different methods and from different host species (see Fig. S3 in the supplemental material), though SS-pLL did not cause significant upregulation of CD86. Control phagocytosable or nonphagocytosable particles did not have an impact on CD86 or CD40 expression (see Fig. S3 in the supplemental material). Similar to the effects on cytokine responses, the effects of pLL on cell surface molecules were absent in the presence of cytochalasin D, which by itself had effects similar to those of pLL (see Fig. S4 in the supplemental material). CD86 upregulation and inhibition of TLR agonist-induced CD40 upregulation were also clearly observed in pLL-exposed BMDMs (data not shown).

**FIG 7:**
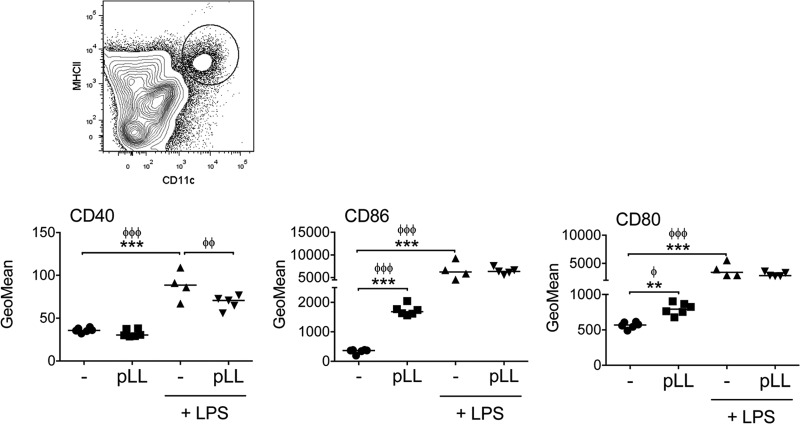
pLL induces upregulation of CD86 and interferes with upregulation of CD40 in DCs *in vivo*. C57BL/6 mice were injected i.p. with FD-pLL, LPS, both stimuli, or vehicle (PBS) only. Eighteen hours later, peritoneal DCs (defined as CD11^+^ MHC-II^+^ cells; see the contour plot at the top and Fig. S5 in the supplemental material) were analyzed for expression of surface molecules. Geometric means of fluorescence intensity were subtracted from the values of the corresponding isotype controls and log transformed for statistical analysis. ANOVA gave overall *P* values of <0.0001. The data shown are from 1 experiment representative of 3 independent experiments; interexperiment statistics are shown (ϕϕϕ, *P* < 0.001; ϕϕ, *P* < 0.01; ϕ, *P* < 0.05), along with intraexperiment ones. ***, *P* < 0.001; **, *P* < 0.01.

In order to obtain an *in vivo* correlate of these *in vitro* data, mice were injected i.p. with pLL and/or LPS, and expression of costimulatory molecules was assessed 18 h later in peritoneal DCs (see Fig. S5 in the supplemental material for the gating strategy). Very similar to what was seen *in vitro*, DCs from mice inoculated with pLL showed clear upregulation of CD86 but no upregulation of CD40; this was accompanied by weak upregulation of CD80 that was not robust from one experiment to the other (Fig. 7). Upon coinjection with LPS, pLL partially inhibited the upregulation of CD40 in DCs without affecting the upregulation of CD80 and CD86 (Fig. 7). As the sole stimulus, pLL gave rise to a nonsignificant trend toward an increase in the number and percentage of peritoneal DCs after 18 h; together with LPS, it did not alter the decrease in DC numbers and percentages caused by this inflammatory stimulus (data not shown).

Thus, DCs exposed to pLL upregulated CD86 selectively both *in vitro* and *in vivo*. Additionally, pLL enhanced TLR-stimulated upregulation of CD86 while inhibiting CD40. Thus, in both the presence and absence of TLR agonists, pLL conditions DCs to adopt semimature phenotypes.

### The capacity of pLL to condition BMDCs is not due to host Igs.

**FIG 8:**
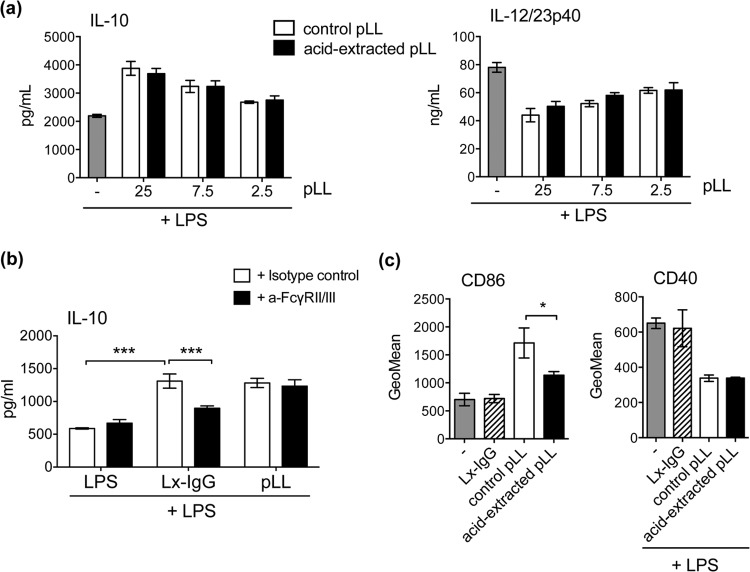
The effects of pLL are not due to host Igs. BMDCs were exposed to the materials indicated, and CD86 expression was measured, or they were further stimulated with LPS as indicated, and cytokines in the supernatants or CD40 expression was measured. (a) IL-10 and IL-12/23p40 after exposure to the indicated doses of pLL (μg), previously subjected or not to acid extraction to release host Igs. (b) IL-10 after exposure to pLL (25 μg) or IgG-coated latex beads (Lx-IgG) (225 μg), with either stimulus having been added after preincubation of the cells with FcγR-blocking antibody or isotype control. BSA-coated latex beads did not cause potentiation of IL-10 secretion (data not shown), showing that the effect of Lx-IgG is indeed due to FcγR cross-linking. (c) CD86 and CD40 after exposure to pLL (25 μg), previously subjected or not to acid extraction or to Lx-IgG (25 μg). None of the materials tested induced cytokines or CD40 upregulation in the absence of LPS. FD-pLL, from a mouse host, was used throughout. The error bars indicate the SD of triplicate (cytokines) or duplicate (surface molecules) wells. ANOVA gave overall *P* values of <0.0001 (a) and <0.01 (b and c). Significant differences caused by acid extraction of pLL or Fc receptor blocking are indicated with asterisks; significant differences caused by the presence of untreated pLL are not indicated. The results shown are representative of between 3 and 6 independent experiments. ***, *P* < 0.001; *, *P* < 0.05.

In spite of the extensive washes with high-ionic-strength solution employed during its preparation, pLL contains host-derived proteins strongly bound to the LL *in vivo*, mostly Igs. As Fc receptor γ (FcRγ) cross-linking is known to potentiate IL-10 and inhibit IL-12 secretion in macrophages (30, 31), it was important to determine if host Igs contributed to the activation phenotype induced by pLL. Extraction with an acid (pH 2) buffer removed well over 90% of the host Igs from pLL of mouse origin without altering its particulate structure (see Fig. S6a and b in the supplemental material). This harsh treatment did not impair the effects of pLL on IL-10 and IL-12/23p40 secretion in LPS-stimulated BMDCs (Fig. 8a). Although the potentiation of IL-10 expression has been previously reported not to be operative in BMDCs (unlike BMDMs) (32), we did observe enhanced IL-10 production by BMDCs responding to high doses of latex beads coated with polyclonal human IgG (Fig. 8b; see Fig. S7a in the supplemental material). This enhancement was diminished by preincubation of cells with a blocking antibody to FcγRII/III (CD16/CD32). In contrast, the effects of pLL (of either mouse or bovine host origin) were not affected by the blocking antibody (Fig. 8b; see Fig. S7a in the supplemental material). Further, addition of cytochalasin D had contrasting effects on the potentiation of IL-10 by pLL or IgG: while, as previously shown, the actin inhibitor caused pLL to have no effect, it caused IgG-coated beads to give rise to much stronger potentiation (see Fig. S7b in the supplemental material). In terms of the capacity to cause CD86 upregulation, extraction of pLL with pH 2 buffer caused a significant decrease in activity (Fig. 8c). However, and similar to the data for IL-10, while, as shown in Fig. S4 in the supplemental material, pLL had positive effects on CD86 expression only in the absence of cytochalasin D, addition of the inhibitor allowed IgG-coated latex beads to strongly potentiate CD86 upregulation by LPS (data not shown). The parasite material's capacity to inhibit CD40 upregulation by LPS was not affected by pH 2 extraction and was also not imitated by IgG-coated latex beads (Fig. 8c).

These results indicate that host Igs are not responsible for the capacity of pLL to influence BMDC activation. Together with the observation that pLL biases BMDC cytokine responses and costimulatory molecule expression independently of the host species origin of the preparation, the data strongly suggest that the LL is intrinsically capable of influencing DC activation independently of adsorbed host proteins.

### The capacity of pLL to bias DC activation is susceptible to reduction of disulfides, but not to oxidation of carbohydrates.

**FIG 9:**
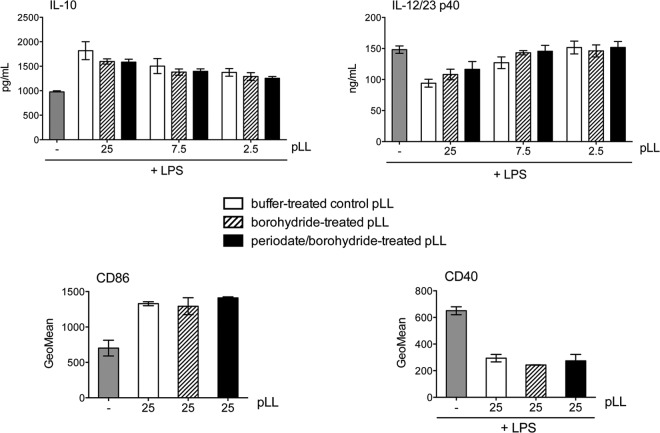
The activity of pLL is not affected by carbohydrate oxidation. BMDCs were exposed to the indicated doses of FD-pLL (μg), previously subjected to oxidation of carbohydrates with sodium periodate followed by reduction of aldehydes with sodium borohydride; to a mock treatment consisting of reduction with borohydride only; or to incubation with neutral buffer. The cells were stimulated or not with LPS, as indicated, and CD86 and CD40 expression and IL-10 and IL-12/23p40 in supernatants were measured. None of the preparations induced cytokines or CD40 upregulation in the absence of LPS. The error bars correspond to SD of triplicate (cytokines) or duplicate (surface molecules) wells. ANOVA gave overall *P* values of <0.0001 (cytokines), <0.001 (CD86), and <0.01 (CD40). Significant differences caused by the presence of untreated or treated pLL (in comparison with medium alone or LPS alone) are not indicated. The results shown are representative of 4 independent experiments.

As mentioned previously, the capacity of pLL to bias BMDC phenotype was insensitive to organic solvents. Together with the observation of only marginal sensitivity to incubation at 100°C (for 1 h) (data not shown), this suggested that the activity of pLL is independent of globular proteins. This fitted our expectation, as by far the major exposed molecular moieties in pLL are the mucin glycans (2, 3, 8). However, oxidation with sodium periodate did not have an effect on the capacity of pLL to bias BMDC cytokine responses or costimulatory molecule expression in comparison to the mock treatment (Fig. 9, compare periodate/borohydride-treated pLL with borohydride-treated pLL). Similar results were obtained with BMDMs (data not shown). In contrast, periodate treatment abrogated the capacity of pLL to be recognized by the plant lectins PNA and RCA1 (Fig. 1b; see Fig. S8 in the supplemental material). Thus, the native carbohydrate structure appears not to be crucial for the effects of pLL on DCs.

**FIG 10:**
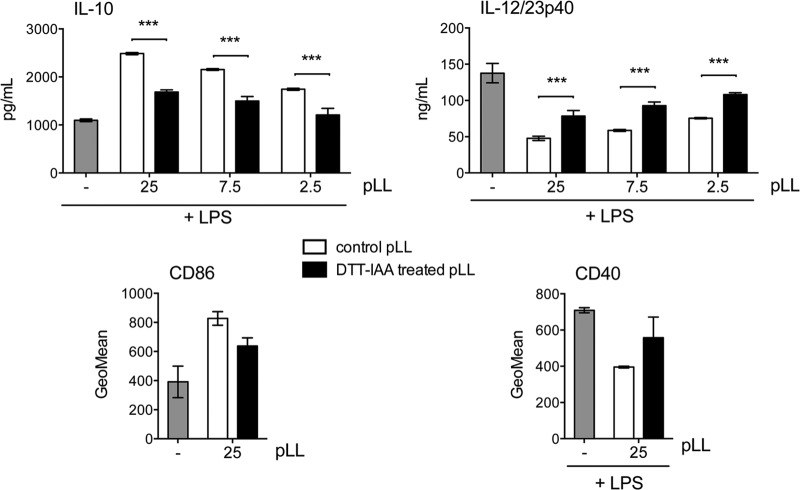
The effects of pLL are susceptible to disulfide reduction. BMDCs were exposed to the indicated doses of FD-pLL (μg), previously treated with DTT to reduce disulfide bonds followed by IAA to block the thiols formed (DTT/IAA-treated pLL), or incubated in buffer only (control pLL). Then, CD86 expression was measured or the cells were additionally stimulated with LPS, and CD40 expression or IL-10 and IL-12/23p40 in supernatants were measured. None of the preparations induced cytokines or CD40 upregulation in the absence of LPS. The error bars correspond to SD of triplicate (cytokines) or duplicate (surface molecules) wells. ANOVA gave overall *P* values of <0.0001 (cytokines) or <0.05 (surface molecules). Significant differences caused by disulfide reduction applied to pLL are indicated with asterisks; significant differences caused by the presence of untreated pLL are not indicated. The results shown are representative of at least 4 independent experiments. ***, *P* < 0.001.

Given the results described above, we next explored the requirement for protein components for the activity of pLL on DCs. Nonspecific digestion of pLL with pronase eliminated all the conventional proteins present in pLL detectable on SDS-PAGE (including host Ig) without altering the material's physical presentation (see Fig. S6a and b in the supplemental material). This treatment caused a trend toward lower capacity to potentiate IL-10, which did not reach statistical significance, and it did not affect the capacity to inhibit IL-12/23p40 (data not shown; see Fig. S6c in the supplemental material). Conventional SDS-PAGE does not allow detection of the major structural components of the LL, i.e., the mucins (7). These mucins are mostly insensitive to pronase treatment (7), probably as a result of the dense glycosylation they bear (11, 12). However, we have previously observed that pronase treatment has a discernible, albeit weak, effect on LL structure, and a similar but stronger effect is brought about by reduction of disulfide bonds using DTT (7). We interpret these results in terms of DTT (and, less efficiently, pronase) targeting a potential disulfide bond formed by an unpaired cysteine residue present in the short nonglycosylated N-terminal extension of one of the LL apomucins (11, 12). We thus assayed the effect of disulfide reduction on the effects of pLL on DCs. Disulfide reduction significantly diminished the capacity of pLL to potentiate IL-10 and inhibit IL-12/23p40; it also gave rise to a clear trend toward diminished capacities to induce CD86 and to inhibit CD40 upregulation (Fig. 10). Complete abrogation of the effects of pLL on BMDCs was not achieved with any of several variants of the treatment tested or by combinations of DTT and pronase treatment (data not shown). Neither pronase digestion (see Fig. S6c in the supplemental material) nor disulfide reduction (see Fig. 1) caused gross alteration in the particle size or appearance of pLL under the light microscope. The fact that disulfide reduction diminishes the effect of pLL on BMDCs probably explains the observation that borohydride treatment *per se* (i.e., when used as a mock treatment, separately from periodate oxidation) tended to diminish the capacity of pLL to affect the BMDC phenotype (Fig. 9, compare buffer-treated pLL and borohydride-treated pLL). Indeed, borohydride can affect proteins through the reduction of disulfide bonds (33).

In summary, pLL conditions BMDCs independently of its carbohydrates and in a manner that requires intact disulfide bonds in the material for full potency.

## DISCUSSION

The ability to inhibit the expression of proinflammatory cytokines and costimulatory molecules induced by TLR agonists in DCs or macrophages appears to be a common feature of helminth parasites or their products (34–40). In this sense, the inhibition of TLR agonist-induced expression of IL-12 and CD40 caused by pLL in BMDCs is expected. The effects of helminth preparations on TLR-triggered production of IL-10 range from inhibitory to potentiating, depending on the helminth extract, TLR agonist, and DC model/species (34, 36, 41–44). IL-10 is induced by TLR agonists with delayed kinetics with respect to proinflammatory cytokines as part of a negative-feedback mechanism (45). IL-10 triggered by TLR engagement can also be enhanced through other signaling pathways (46–48). The pattern of response of BMDCs to pLL suggests analogous modulation of NF-κB-dependent responses without NF-κB activation. That exposure to pLL as the sole stimulus does not cause NF-κB activation is strongly suggested by the absence of response in cytokines known to be dependent on this signaling modulus. In this context, what stands out is the observation that pLL on its own induces, *in vitro* and *in vivo*, the upregulation of CD86. Selective upregulation of CD86 among major costimulatory molecules was also reported in DCs exposed to the excretory-secretory products of the nematode Nippostrongylus brasiliensis (49). However, in contrast to our experimental system, this was accompanied by secretion of IL-6 and IL-12/23p40, suggesting that exposure to the N. brasiliensis extract caused some NF-κB activation. It is tempting to speculate that contact with pLL induces in myeloid cells an NF-κB-independent pathway leading to CD86 upregulation. Whatever the pathways engaged in DCs are, they appear to be shared with macrophages, as bone marrow-derived macrophages exposed to pLL reacted very similarly to BMDCs in regard to IL-12, IL-10, TNF-α, IL-6, CD80, CD86, and CD40 (data not shown).

Somewhat surprisingly, the influence of pLL on BMDC activation appeared to be independent of the carbohydrates present in the material. It is thus conceivable that the LL carbohydrates may be inert with respect to systemic innate immunity (although interaction with liver-specific lectins must be considered [3, 50]). The activity of pLL on myeloid cells was not due to bound host Igs; it was, however, strongly diminished by the reduction of disulfide bonds. The N-terminal extension of the second most highly expressed LL apomucin subfamily (EGC04254/EGC02904 or EgrG_002012280, according to transcriptome or genome annotation, respectively [11, 12]; GenBank accession no. CDI70203.1) bears what appears to be the only cysteine residue present in the LL apomucins together. We speculate that this residue may form a disulfide bond between two apomucin molecules and that it is this disulfide that is targeted by dithiothreitol (or borohydride) treatment of pLL. Thus, engagement of a cellular receptor by the disulfide-dimerized N terminus of apomucin EgrG_002012280 is an interesting possibility. On the other hand, as complete abrogation of the effects of pLL on DCs by disulfide reduction, either alone or in combination with proteolysis, was not observed, a more complex type of interaction must also be considered. DCs and macrophages are now recognized to decode essentially all aspects of particulate materials they encounter, including size, shape, rigidity, and microtopography (51). Recognition of pLL as a material would be in agreement with the lack of capacity of soluble LL preparations to alter macrophage responses to LPS (reference 23 and our unpublished results). The effects of pLL on DCs evidently require direct contact. The effects may also need particle internalization, as suggested by experiments using cytochalasin D. However, further work is needed on this point, as examples exist of DC responses sensitive to the inhibitor that apparently depend only on events at the cell surface (52). Also, as the effects of pLL and those of cytochalasin D *per se* were qualitatively similar, the possibility that the parasite material elicits signals that have an impact on the actin cytoskeleton so that its effects and those of cytochalasin D are redundant cannot be ruled out.

The activation phenotype of BMDCs conditioned by pLL, either on its own (CD86^med^ CD80^lo^ CD40^−^ IL-12^−^ IL-10^−^) or in the additional presence of TLR agonists (CD86^hi^ CD80^hi^ CD40^lo^ IL-12^lo^ IL-10^hi^), fall within what has previously been termed “semimature” DCs (53). Such DCs are generally associated with tolerogenesis, and in particular with the induction of the Tr1 subtype (FoxP3^−^ IL-10^+^) of regulatory T cells (54). Although the definition of semimature DCs is fairly broad, the low or absent expression of CD40 in particular is strongly associated with tolerogenicity (55, 56). The next stage of these studies will determine the impact of pLL on the ability of DCs to activate T cells.

The results we present in this work are relevant, in the infection context, to cells encountering materials shed from the LL. In experimental (BALB/c) mouse infection, both spleen and blood DCs (defined as CD11c^+^ cells) and macrophages (defined as CD11b^+^ cells) were observed to express CD86 at levels above those of controls (6); CD40 levels have not been studied, and neither has the phenotype of myeloid cells near the parasite itself. We envisage that the DC phenotype in larval Echinococcus infections must be influenced both by LL-derived materials and by soluble, secreted mediators. The effects of E. granulosus metacestode secretions on DCs have not been studied. Cyst fluid, which is normally not exposed to the host, causes upregulation of activation markers, production of inflammatory cytokines, and NF-κB activation on human differentiated monocyte-derived DCs (57, 58). BMDC responses to *in vitro* metacestode secretions have been studied for E. multilocularis (41). The cells do not respond with cytokines and downregulate MHC-II and CD86 below basal levels; upon subsequent challenge with LPS, they show impaired MHC-II, CD86, IL-12p70, and IL-10 responses. These observations are in broad agreement with the phenotype observed for local DCs in E. multilocularis experimental infection (59, 60). Our current study provides a platform for future work addressing DC conditioning by E. granulosus metacestode secretions, as well as examining the phenotype of DCs in contact with the larval parasite in infection.

In addition to its role in E. granulosus infection, conditioning of DCs by pLL is interesting in its own right. pLL is a relatively simple material that retains its capacity to condition DCs after heat treatment and oxidation of its major carbohydrate component and even keeps most of this ability after extensive proteolysis. Particles made from synthetic biomaterials destined for tissue engineering generally elicit proinflammatory cytokines from DCs and, in consequence, display adjuvanticity toward adsorbed proteins (61). Thus, mimicking the relevant surface properties of pLL in synthetic biomaterials is an intriguing possibility.

## Supplementary Material

Supplemental material
