# Supplementary material for: Unconventional Maturation of Dendritic Cells Induced by Particles from the Laminated Layer of Larval Echinococcus granulosus
Source: Infect Immun. 2014 Aug;82(8):3164–76. doi: 10.1128/IAI.01959-14 (PMC4136206; doi:10.1128/IAI.01959-14)
Supplement: Supplemental material [file supp_82_8_3164__index.html]

Unconventional Maturation of Dendritic Cells Induced by Particles from the Laminated Layer of Larval Echinococcus granulosus — Supplemental material 

# Unconventional Maturation of Dendritic Cells Induced by Particles from the Laminated Layer of Larval Echinococcus granulosus

## Supplemental material

**Files in this Data Supplement:**

- Supplemental file 1 -

  Fig. S1. Exposure of BMDCs to high doses of pLL causes death in a small proportion of cells. Fig. S2. pLL potentiates IL-10 and IL-12/23p40 responses to LPS *in vivo*, without inducing these cytokines *per se*. Fig. S3. pLL causes up-regulation of CD86 and inhibits CD40 up-regulation in BMDCs irrespective of the host species origin of the material or the pulverization method used to prepare it; control particles do not show the same activities. Fig. S4. The effects of pLL on expression of cell surface markers in BMDCs are not observed in the presence of an actin cytoskeleton inhibitor. Fig. S5. Defining peritoneal dendritic cells as CD11c+ MHCII+ cells or CD11c+ MHCII+ CD19- F4/80- cells produced identical results. Fig. S6. pLL subjected to acid extraction of the Ig content or to pronase digestion has the same physical presentation as control pLL. Fig. S7. The effects of pLL from bovine host on IL-10 are not due to host immunoglobulins. Fig. S8. Periodate treatment abrogates binding of plant lectins to pLL.

  PDF, 415K
