# Supplementary material for: Unconventional Maturation of Dendritic Cells Induced by Particles from the Laminated Layer of Larval Echinococcus granulosus
Source: Infect Immun. 2014 Aug;82(8):3164–76. doi: 10.1128/IAI.01959-14 (PMC4136206; doi:10.1128/IAI.01959-14)
Supplement: Supplemental material [file IAI.01959-14_zii999090779so1.pdf]

SUPPLEMENTAL FIGURE LEGENDS

Figure S1. Exposure of BMDCs to high doses of pLL causes death in a small proportion of cells. Cells were incubated for 18 h with the indicated doses of FD-pLL, alone or followed by LPS, as indicated. Cell viability was analysed with the help of the Live/dead™ dye. The results shown are mean +/- SD of 3 independent experiments. The ANOVA analysis gave an overall p value < 0,0001. Asterisks denote significant differences with respect to incubation in the absence of pLL. Slight reduction in DC viability was also indicated by results obtained using the MTT assay (not shown).

Figure S2. pLL potentiates IL-10 and IL-12/23p40 responses to LPS in vivo, without inducing these cytokines *per se*. C57BL/6 mice were injected i.p. with FD-pLL, LPS, the two stimuli together, or vehicle (PBS). Three h later, cytokines were measured in the peritoneal lavage. ANOVA analyses gave overall p values < 0.0001. The data shown are from one experiment representative of 2 independent experiments. For IL-10, a similar result was obtained 18 h after injection, but with much lower overall cytokine levels (data not shown). At this time point no significant differences were observed for IL-12/23p40, which was present at extremely low levels.

Figure S3. pLL causes up-regulation of CD86 and inhibits CD40 up-regulation in BMDCs irrespective of the host species origin of the material or the pulverisation method used to prepare it; control particles do not show the same activities. BMDCs were exposed to FD-pLL made from bovine origin material, to FD-pLL or SS-pLL, both from mouse origin material, or to control phagocytosable (0.8-µm BSA-coated latex beads; "Lx") or non-phagocytosable particles (Sephadex G-100; "G-100"); all materials were

tested at 25 μg total dry mass per million cells. One hour later, cells were stimulated or not with LPS as indicated and 18 h later analyzed for CD86 and CD40 expression. None of the materials tested caused up-regulation of CD40 in the absence of LPS. ANOVA analyses gave overall p values smaller than 0.0001 for both CD86 and CD40. Bars indicate the SD of duplicate wells. Asterisks denote differences with respect to incubation in medium (CD86) or with stimulation with LPS alone (CD40). The results shown are representative of 4 independent experiments.

Figure S4. The effects of pLL on the expression of cell surface markers in BMDCs are not observed in the presence of an actin cytoskeleton inhibitor. BMDCs were exposed to FD-pLL (25 μg), stimulated or not with LPS, and 18 h later analyzed for cell surface markers in the absence or presence of cytochalasin D. Bars indicate SD of triplicate wells. ANOVA analyses gave overall p values < 0.0001 (CD86) and < 0.001 (CD40). Asterisks denote differences with respect to incubation in medium (CD86) or with stimulation with LPS alone (CD40). Results shown are representative of 3 independent experiments.

Figure S5. Defining peritoneal dendritic cells as CD11c^{+} MHCII^{+} cells or CD11c^{+} MHCII^{+} CD19^{-} F4/80^{-} cells produced identical results. Since in our samples we found no CD11c^{+} MHCII^{+} cells that were F4/80^{+} and/or CD19^{+}, the two last markers were dispensable for defining DCs. Accordingly, measured DC expression of CD86, CD40 and CD80 was identical irrespective of which way DCs were defined (overlaid histograms on the right).

Figure S6. pLL subjected to acid extraction of the Ig content or to pronase digestion has the same physical presentation as control pLL. FD-pLL was treated with PBS

(control), with glycine-HCl pH 2.0 buffer, or with pronase. Afterwards, all remaining non-structural proteins were extracted using SDS-PAGE sample buffer and analyzed by SDS-PAGE (reducing conditions) followed by Coommassie staining **(a)**. Each lane corresponds to 1 mg of pLL total dry mass; IgG and IgM heavy chain (H) and Ig light chain (L) bands were identified on the basis of their characteristic migration. The same samples were analysed by fluorescence microscopy, after staining of the mucin glycans with PNA-FITC **(b)**; the host species origins of materials are indicated in each case; a fluorescence microscopy image of control bovine pLL has already been shown in Fig. 1. In addition, FD-pLL treated with pronase or buffer control were analysed for influence on IL-10 production by BMDC stimulated with LPS **(c)**. Bars correspond to SD of triplicate wells. ANOVA analysis gave overall p value < 0.01. Significant differences caused by the presence of untreated pLL are not indicated. The result is representative of 6 independent experiments.

Figure S7. The effects of pLL from bovine host on IL-10 are not due to host immunoglobulins. BMDCs were exposed to FD-pLL (25 μg) or IgG-coated latex beads (Lx-IgG; 75 μg) plus LPS, in the absence or presence of FcγRII/III blocking antibodies **(a)** or cytochalasin D **(b)**, and supernatants analyzed 18 h later for IL-10. Bars correspond to SD of triplicate wells. Anova analyses gave overall p values < 0.0001. The difference between addition of FcγRII/III blocking antibody and isotype control for Lx-IgG had a p value = 0.054. BSA-coated latex beads did not cause potentiation of IL-10 secretion (data not shown). The absence of effect of FcγRII/III blocking antibody on potentiation of IL-10 induced by pLL was observed in 3 independent experiments. The absence of potentiation of IL-10 response by pLL in the presence of cytochalasin D and the potentiation of IL-10 by

IgG-coated beads in the presence of the inhibitor were observed in at least two independent experiments.

Figure S8. Periodate treatment abrogates binding of plant lectins to pLL. FD-pLL was treated with sodium periodate followed by reduction of aldehydes with sodium borohydride ("periodate/borohydride-treated pLL"), to a mock treatment consisting of reduction with borohydride only ("borohydride-treated pLL), or to incubation with neutral buffer ("buffer treated control pLL"). The different suspensions were then solubilized by sonication and spotted onto a nitrocellulose membrane using the BioDot equipment (BioDot Inc). After blocking with 0.2 % Tween 20 in PBS, strips were probed with 1 μg/ml of biotin-PNA or RCA I (Vector Labs), followed by streptavidin-peroxidase (Pierce). Development used the Supersignal WestPico chemiluminiscent substrate (Thermo) and signals were quantified using the Quantity One software, and expressed in arbitrary units (AU). Each sample-lectin combination was assayed in triplicate, as shown below; error bars in the plot above correspond to SD of these triplicates.

Figure S1

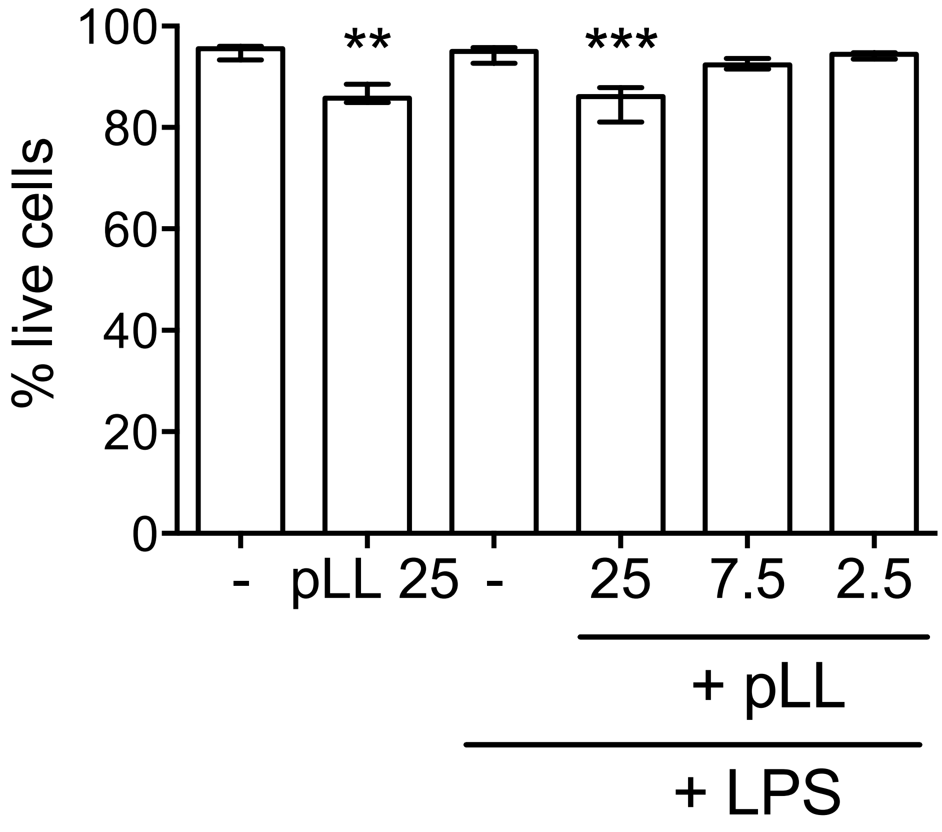

Figure S2

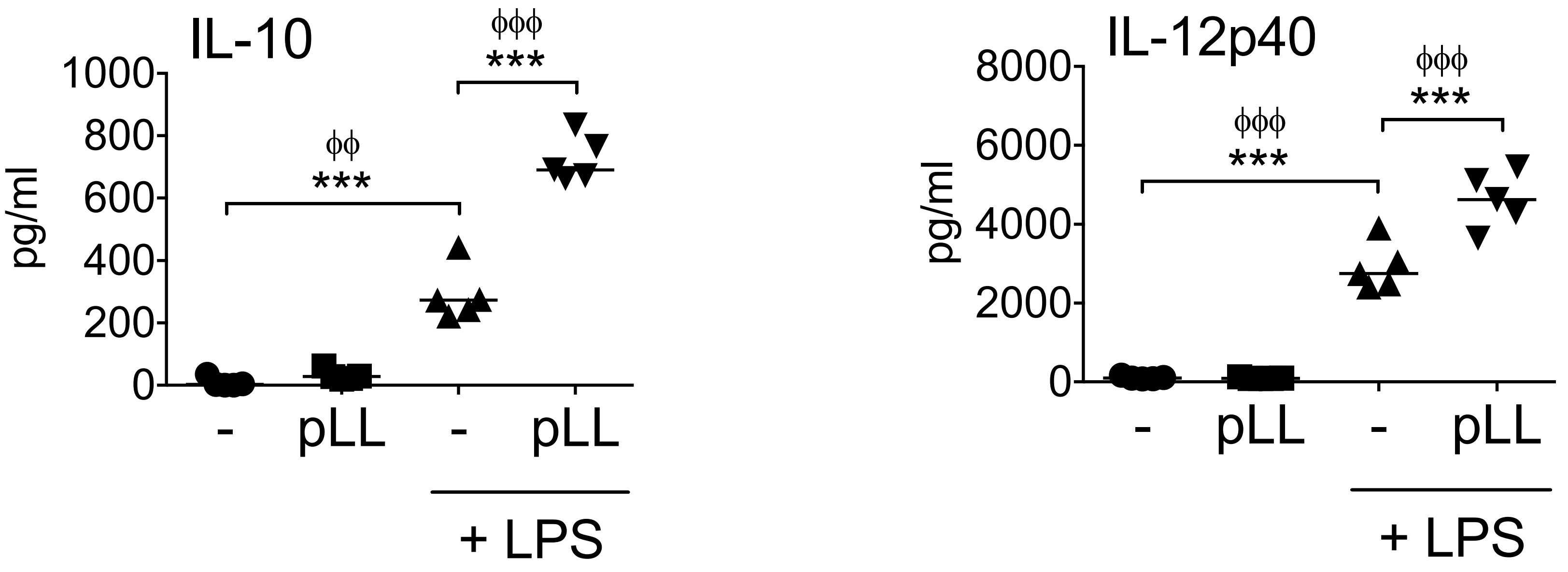
IL-10
1000
800
600
400
200
0
pg/ml
ϕϕϕ
***
ϕϕ
***
-
pLL
-
pLL
+ LPS
IL-12p40
8000
6000
4000
2000
0
pg/ml
ϕϕϕ
***
ϕϕϕ
***
-
pLL
-
pLL
+ LPS

Figure S3

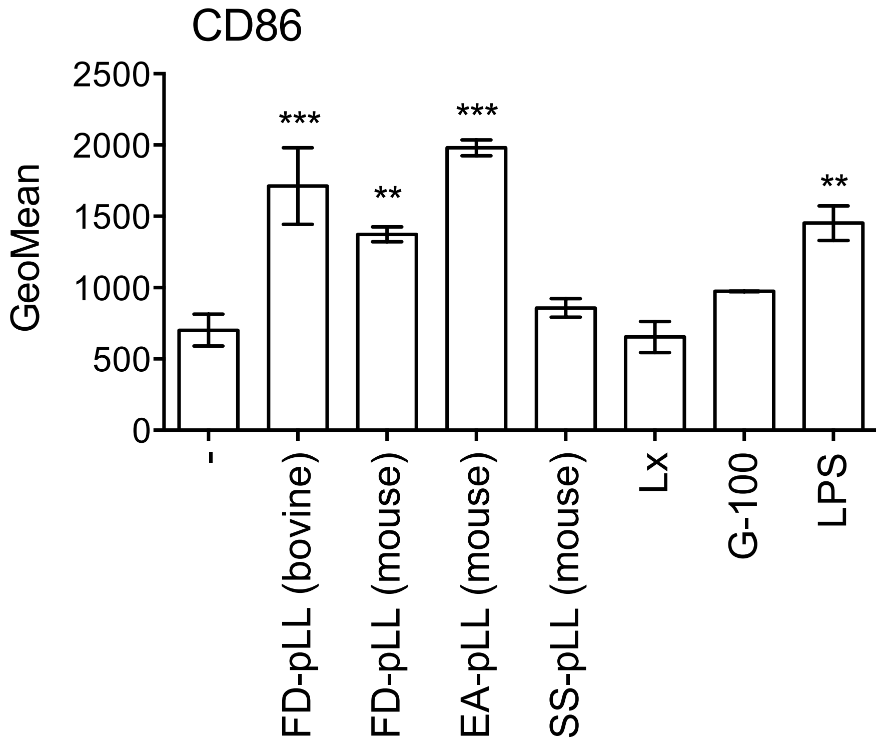

CD86
GeoMean
2500
2000
1500
1000
500
0
***
**
***
**
-
FD-pLL (bovine)
FD-pLL (mouse)
EA-pLL (mouse)
SS-pLL (mouse)
Lx
G-100
LPS

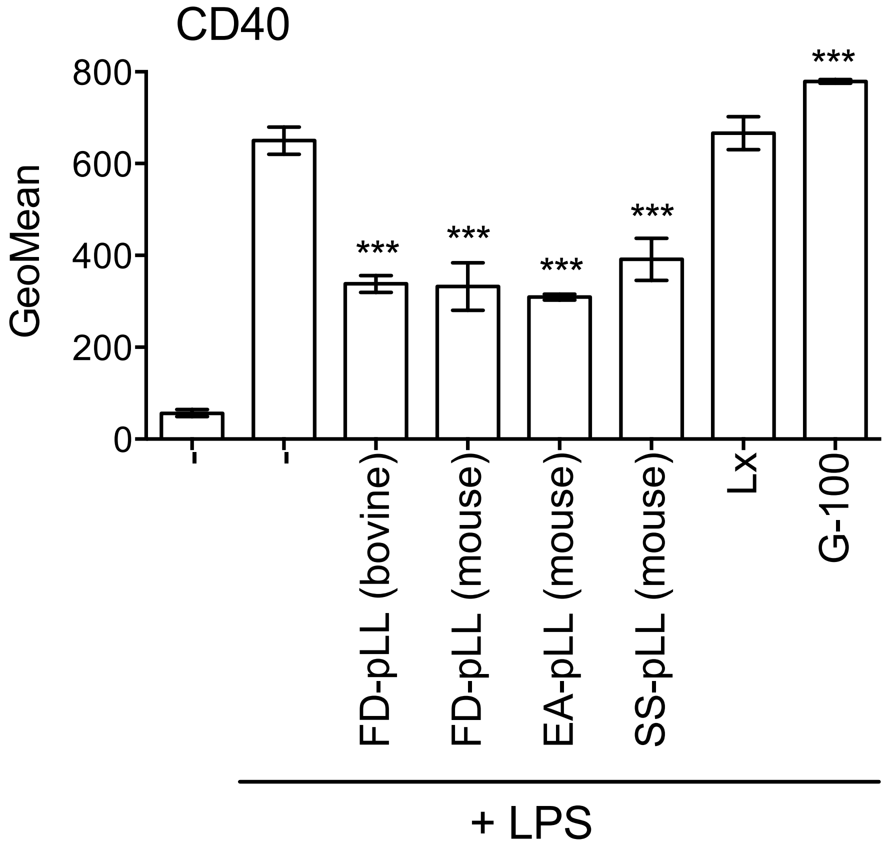

CD40
GeoMean
800
600
400
200
0
***
***
***
***
***
-
-
FD-pLL (bovine)
FD-pLL (mouse)
EA-pLL (mouse)
SS-pLL (mouse)
Lx
G-100
+ LPS

Figure S4

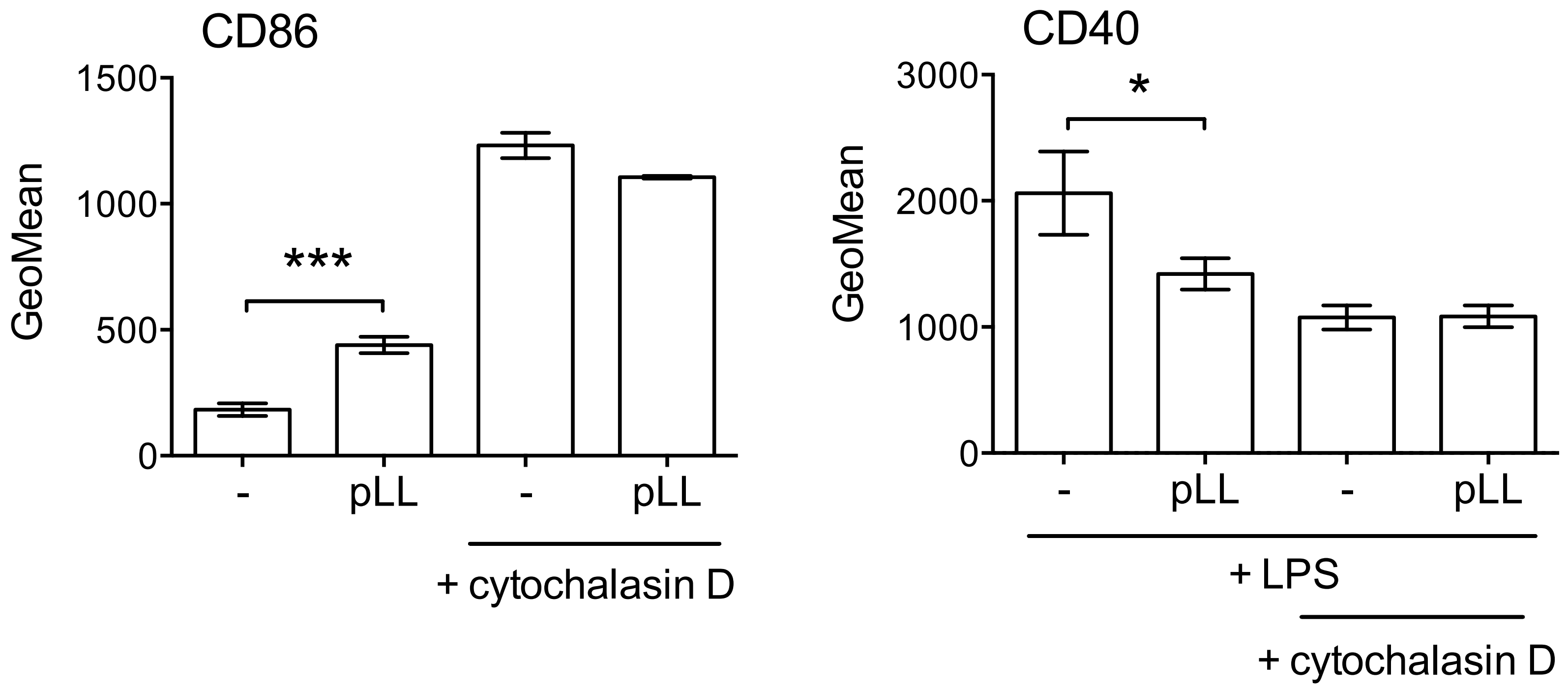
CD86
1500
1000
500
0
GeoMean
***
-
pLL
-
pLL
+ cytochalasin D
CD40
3000
2000
1000
0
GeoMean
*
-
pLL
-
pLL
+ LPS
+ cytochalasin D

Figure S5

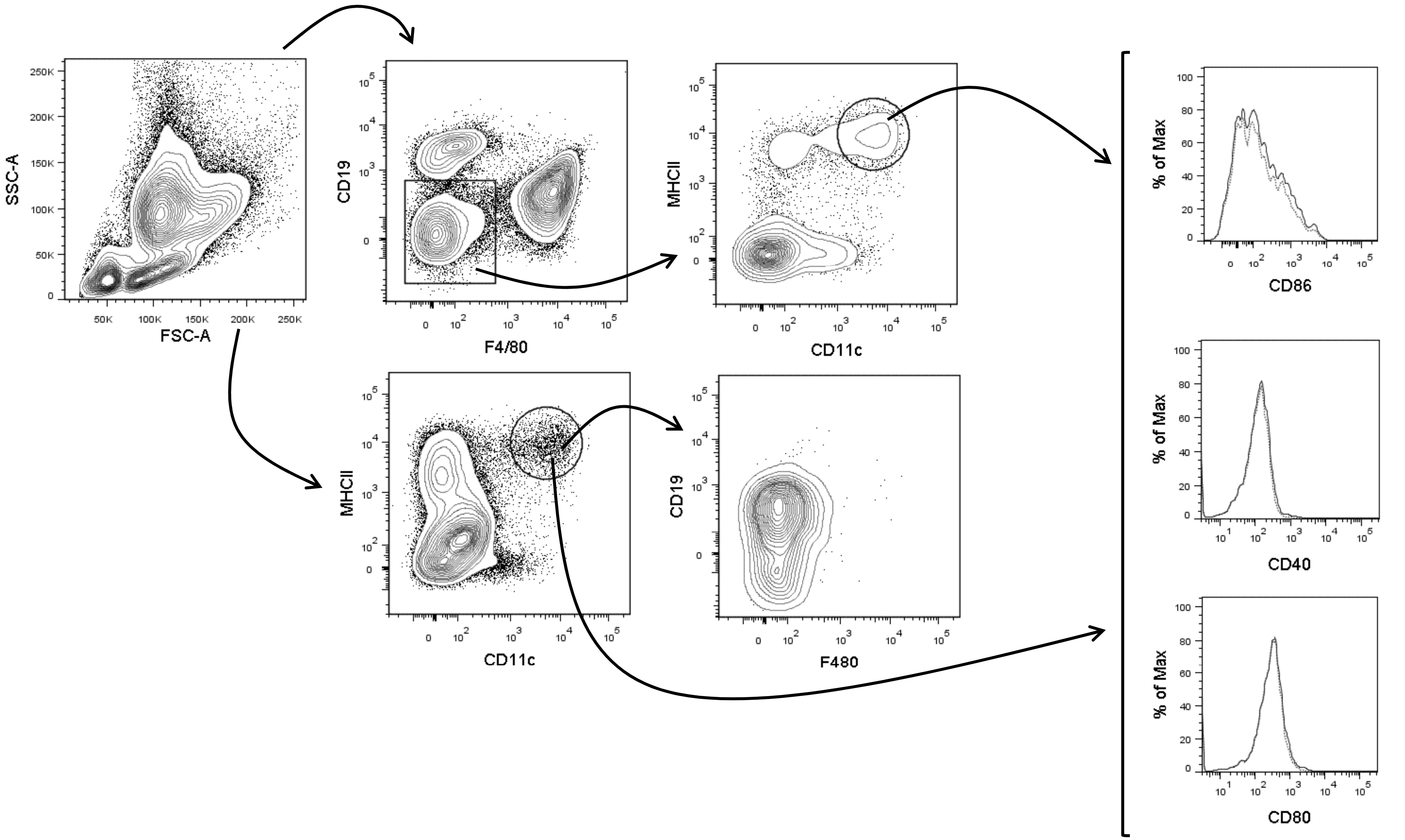

SSC-A
FSC-A
CD19
F4/80
MHCII
CD11c
CD19
F480
MHCII
CD11c
% of Max
CD86
% of Max
CD40
% of Max
CD80

Figure S6

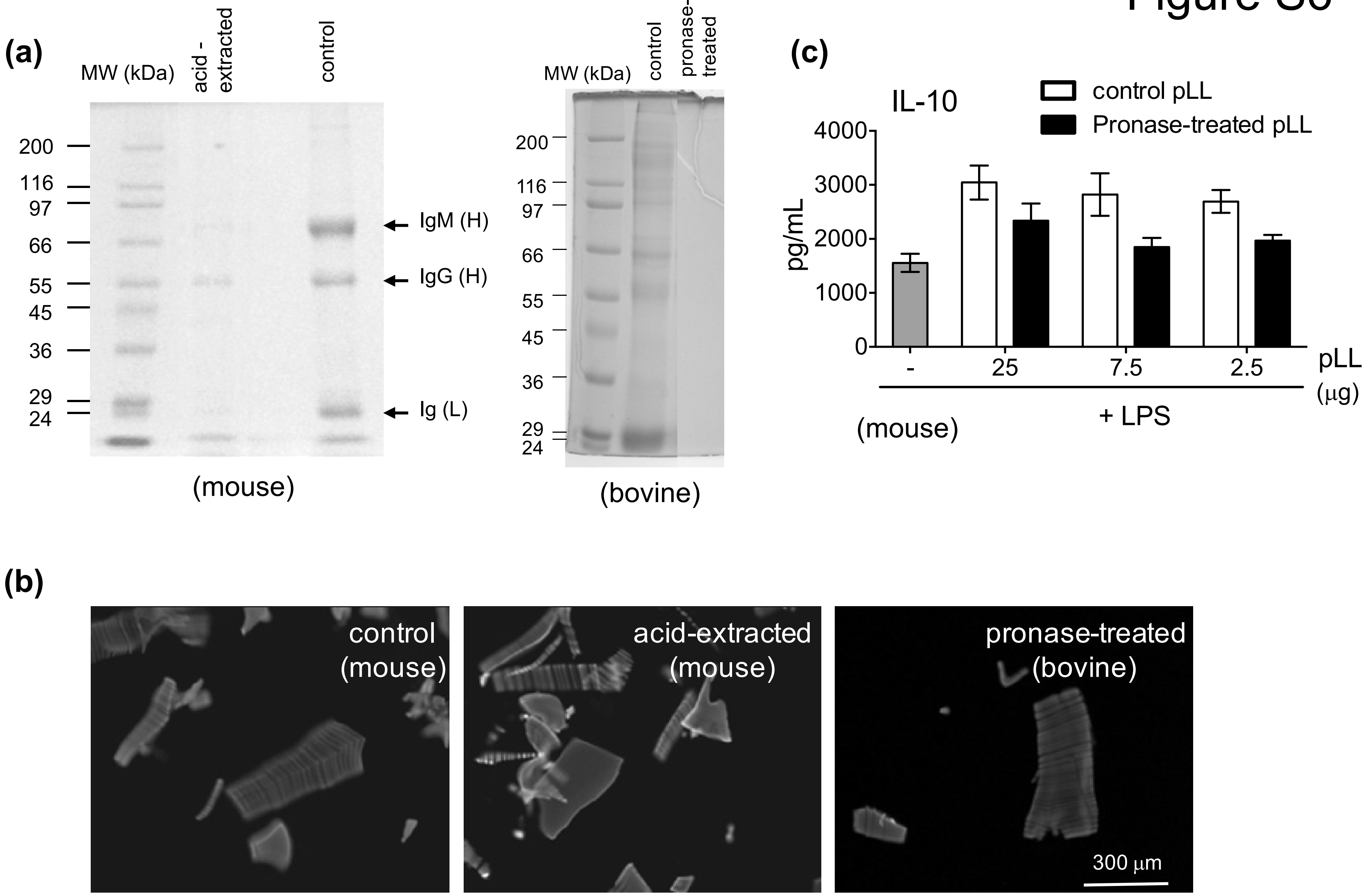
(a)
MW (kDa)
acid - extracted
control
200
116
97
66
55
45
36
29
24
IgM (H)
IgG (H)
Ig (L)
(mouse)
MW (kDa)
control
pronase-treated
200
116
97
66
55
45
36
29
24
(bovine)
(c)
IL-10
control pLL
Pronase-treated pLL
pg/mL
4000
3000
2000
1000
0
-
25
7.5
2.5
pLL (μg)
+ LPS
(mouse)
(b)
control (mouse)
acid-extracted (mouse)
pronase-treated (bovine)
300 μm

Figure S7

(a)

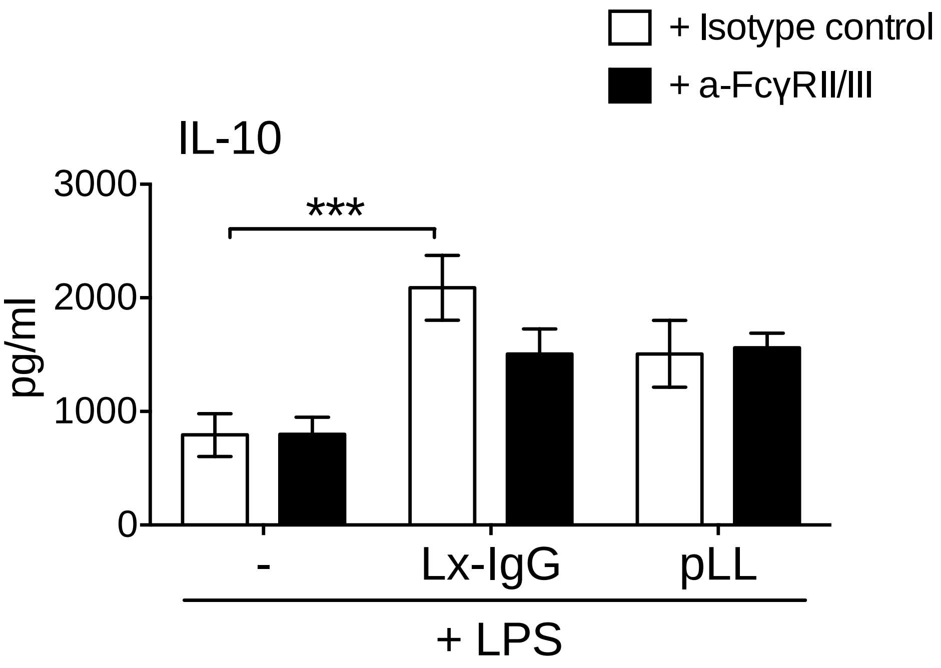
+ Isotype control
+ a-FcγRII/III
IL-10
3000
2000
1000
0
pg/ml
***
-
Lx-IgG
pLL
+ LPS

(b)

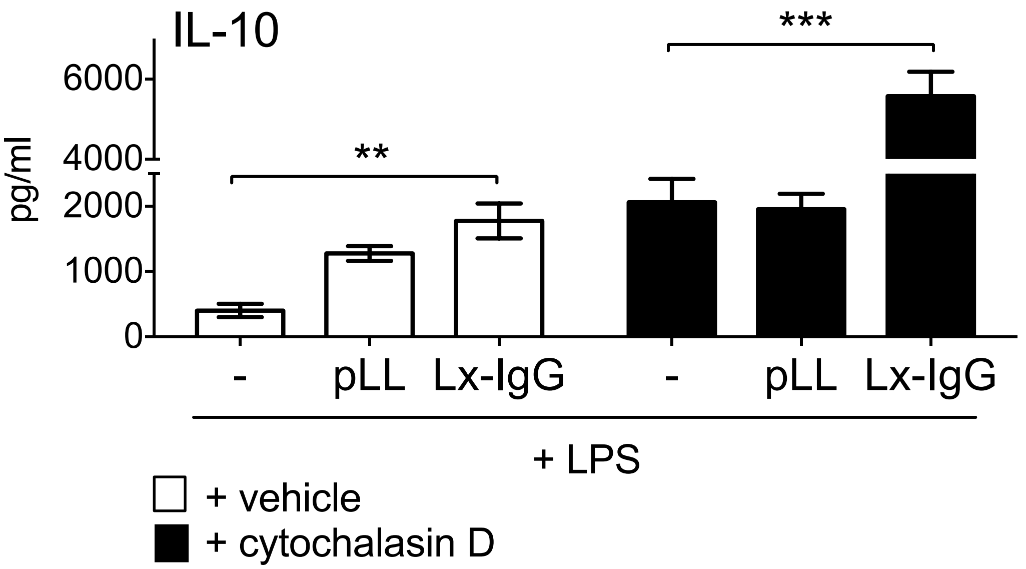
IL-10
6000
4000
2000
1000
0
pg/ml
**
***
-
pLL
Lx-IgG
-
pLL
Lx-IgG
+ LPS
+ vehicle
+ cytochalasin D

Figure S8

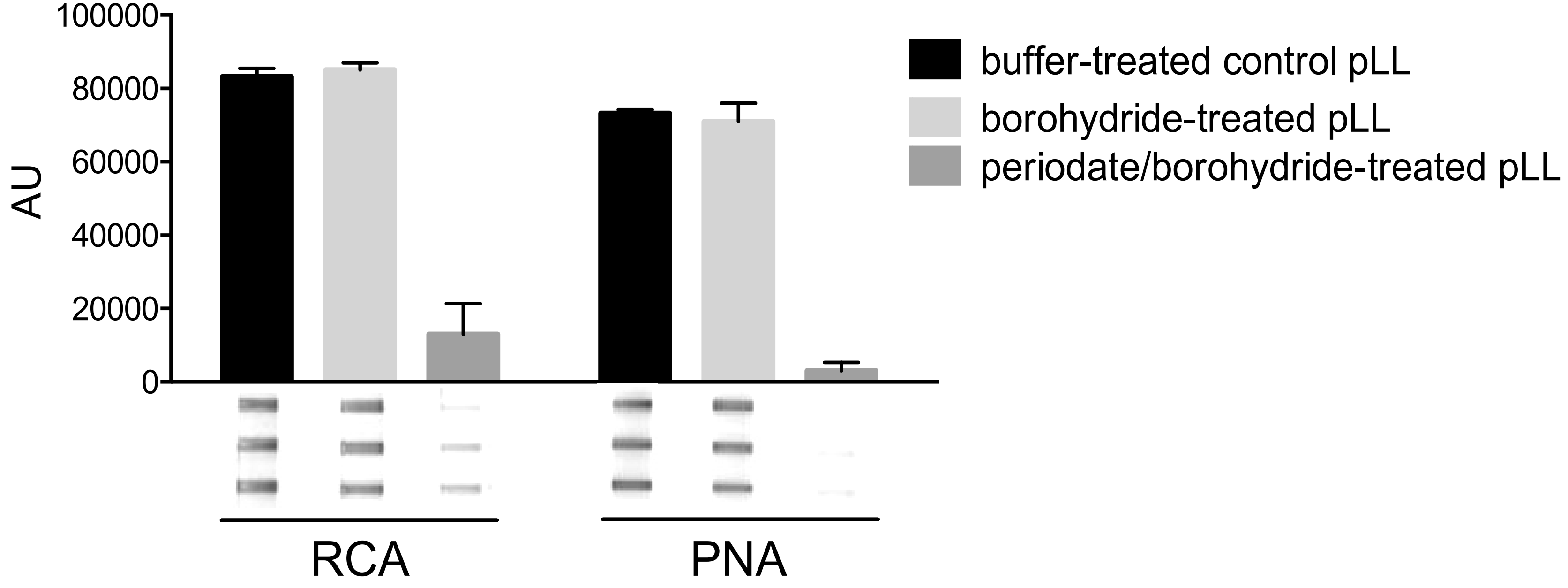

100000
80000
60000
40000
20000
0
AU
buffer-treated control pLL
borohydride-treated pLL
periodate/borohydride-treated pLL
RCA
PNA
